# Report from enhanced safety surveillance of two influenza vaccines (Vaxigrip and Intanza 15 μg) in two European countries during influenza season 2016/17 and comparison with 2015/16 season

**DOI:** 10.1080/21645515.2017.1405882

**Published:** 2017-12-20

**Authors:** Anne Laure Chabanon, Hélène Bricout, Céline Ballandras, Audrey Souverain, Timothy David Caroe, Karina M. Butler

**Affiliations:** a Sanofi Pasteur, Lyon, France; b Sanofi Pasteur MSD, Lyon, France; c Aixial, Boulogne-Billancourt, France; d Lighthouse Medical Practice, Eastbourne, East Sussex, UK; e Our Lady's Children's Hospital Crumlin, Dublin, Republic of Ireland

**Keywords:** Inactivated influenza vaccine, influenza, passive surveillance, The Republic of Ireland, United Kingdom, vaccines and immunization

## Abstract

Passive enhanced safety surveillance (ESS) was implemented in the United Kingdom and in the Republic of Ireland for Vaxigrip and Intanza 15 µg influenza vaccines during the 2016/17 influenza season. Lessons learned during 2015/16 ESS implementation were integrated and applied towards the current ESS. The primary objective was to estimate the reporting rates of suspected adverse reactions (ARs) occurring within 7 days of vaccination with Vaxigrip or Intanza 15 µg. For Vaxigrip (N = 962), 17 vaccinees (1.8%) reported 59 suspected ARs (6.1%) within 7 days of vaccination. For Intanza 15 µg (N = 1000), 21 vaccinees (2.1%) reported 101 (10.1%) suspected ARs within 7 days of vaccination. No obvious pattern in the type of suspected ARs or their frequency was observed for either vaccine. None of the frequencies of suspected ARs were above the 2015/16 ESS frequencies for Vaxigrip, whereas for Intanza 15 µg only one AR (oropharyngeal pain) crossed the historical threshold. There was no change in reactogenicity and data was consistent with the safety profiles of the two vaccines. The passive ESS experience gained from season to season will help to contribute to a sustainable safety surveillance system of seasonal influenza vaccines early in the season.

## Introduction

Since April 2014, enhanced safety surveillance (ESS) is required annually by the European Medicines Agency (EMA) for all seasonal influenza vaccines. Influenza vaccination is the only preventive measure for seasonal influenza and offers protection against two A strains and one or two B strains.^1^ Each year, influenza vaccine composition is updated following the World Health Organization's (WHO) recommendation based on extensive surveillance of influenza strains worldwide to adapt the vaccine composition to the epidemiological situation and provide optimal protection for the population.

The Pharmacovigilance Risk Assessment Committee (PRAC) provided interim guidance on the requirement for a safety surveillance system at the start of each influenza season with the goal to detect any clinically significant change in the frequency or severity of expected reactogenicity (beyond that known or expected with the previous year's vaccine composition) to rapidly identify and mitigate the potential of more serious risks as exposure to the vaccine increases.^2^ Sub-analysis of more than one batch should be conducted to ensure that any increase in reactogenicity observed is due to the product rather than to a specific batch.^2^^,^^3^

To best implement the interim guidance, a continuous dialog between members of the EMA/PRAC/Vaccines Working Party (VWP) and Vaccines Europe Safety Task Force (composed of European influenza manufacturers) has been maintained. Previous years experiences and lessons learned identified paths of improvements. These included the need to improve awareness and stimulate reporting, the need for an increased signal detection early in the season, and the importance of the timely reporting of any potential new signals. The interim guidance revision initially planned in 2016 was put on hold until a more feasible and sustainable ESS strategy could be developed and shown to provide high quality data.

Results of 2015/16 passive ESS for Vaxigrip (intramuscular trivalent split virion inactivated influenza vaccine) and Intanza 15 µg (intradermal trivalent split virion inactivated influenza vaccine) were published previously.^3^ Challenges faced during 2015/16 ESS implementation with respect to study efficiency, age group representation, and site selection were addressed and integrated in the 2016/17 ESS. United Kingdom (UK) and the Republic of Ireland (ROI) selected for conducting the ESS for Vaxigrip and Intanza 15 µg since in these countries vaccine availability was assured with good representation of all age groups targeted for vaccination, thus enabling generation of data early in the season. The primary objective of this ESS was to estimate reporting rates of suspected adverse reactions (ARs) occurring within 7 days following routine vaccination with Vaxigrip or Intanza 15 µg during the 2016/17 influenza season. The secondary objectives were to estimate reporting rates of suspected ARs occurring within 7 days following routine vaccination with Vaxigrip or Intanza 15 µg according to predefined age groups; to estimate the reporting rate of serious suspected ARs at any time following vaccination; and to compare vaccinees' reporting rates (VRR) of suspected ARs observed during the 2016/17 ESS with the VRR of 2015/16 ESS.^3^ Furthermore, if safety signals were detected during the ESS data weekly review, an exploratory objective was to estimate the reporting rate of suspected ARs per batch, whenever possible, to avoid false attribution of the signal to the general intrinsic safety profile of the product.

## Results

### Exposure data

**Figure 1.**
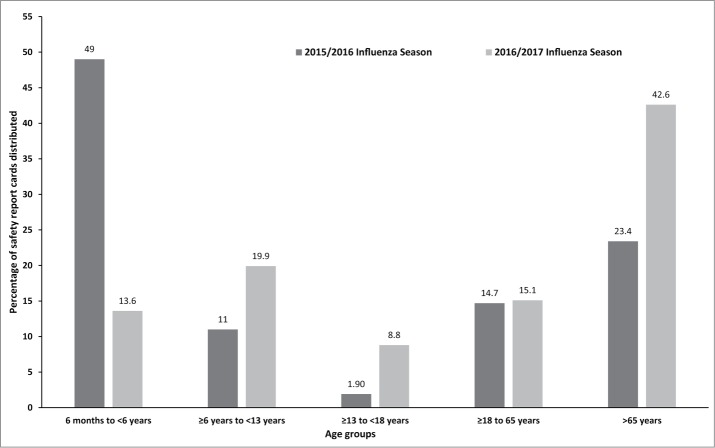
Percentage of Vaxigrip safety report cards distributed during the 2015/16 influenza season and the 2016/17 influenza season by age group. ^a^The total number of safety report cards distributed was 1012 and 962 for the 2015/16 influenza season and the 2016/17 influenza season, respectively.

A total of 962 Vaxigrip safety report cards (SRCs) from 3 sites in the UK and 11 sites in the ROI, and 1000 Intanza 15 µg SRCs from 4 sites in the UK were distributed in 39 and 12 days respectively between 20 September and 28 October 2016. For Vaxigrip, SRC distribution to the pre-defined age groups was compared between the 2016/17 and 2015/16 influenza seasons (Fig. 1). Current ESS had a greater proportion of vaccinees in ≥6 to <13 years, ≥13 to <18 years, and >65 years age groups.

Several different batches of Vaxigrip were used during this ESS. Five batches were administered to ≥100 vaccinees and 14 additional batches to <100 vaccinees. For Intanza 15 µg due to a very fragmented market share, the ESS covered 4 different batches. Since 995 vaccinees received the same vaccine batch, a per batch sub-analysis was impossible. Specific batch analysis was not conducted as exploratory objective since no safety signal was detected.

### Safety data

Seventeen Vaxigrip vaccinees (VRR = 1.8%) reported a total of 59 suspected ARs (AR reporting rate [ARR] = 6.1%), all occurring within 7 days of vaccination. The highest VRR (3.4%) was observed in vaccinees aged ≥18 to 65 years (Table 1). Of the 5 vaccinees in this age group who reported 15 suspected ARs, 3 were women vaccinated while pregnant and who reported as AR “no adverse event”. The highest ARR of 13.0% was observed in 4 vaccinees (VRR = 3.1%) aged 6 months to <6 years. Vaxigrip overall reporting rates for current ESS (1.8%) were in the same range but were lower compared to the 2015/16 ESS overall reporting rates (3.1%) (Table 1).

**Table 1.** Reporting rates of suspected adverse reactions and PRAC adverse reactions of interest occurring within 7 days during 2015/16 and 2016/17 enhanced safety surveillance.

	2015/16 ESS								2016/17 ESS							
	AR reporting rate				Vaccinees reporting rate				AR reporting rate				Vaccinees reporting rate			
	Suspected AR		PRAC AEI		Suspected AR		PRAC AEI		Suspected AR		PRAC AEI		Suspected AR		PRAC AEI	
	n	%	n	%	n	%	n	%	n	%	n	%	n	%	n	%
Vaxigrip																
6 months to <6 years	40	8.1	20	4.0	14	2.8	11	2.2	17	13.0	11	8.4	4	3.1	4	3.1
≥6 to <13 years	8	7.2	7	6.3	2	1.8	2	1.8	10	5.2	8	4.2	3	1.6	3	1.6
≥13 to <18 years	0	0.0	0	0	0	0.0	0	0	0	0.0	0	0.0	0	0.0	0	0
≥18 to 65 years	12	8.1	4	2.7	4	2.7	2	1.3	15	10.3	6	4.1	5	3.4	2	1.4
>65 years	50	21.1	11	4.6	11	4.6	7	3.0	17	4.1	3	0.7	5	1.2	3	0.7
Total Vaxigrip	110	10.9	42	4.2	31	3.1	22	2.2	59	6.1	28	2.9	17	1.8	12	1.2
Intanza 15 µg^a^																
Total Intanza 15 µg	99	9.7	53	5.2	29	2.9	26	2.6	101	10.1	44	4.4	21	2.1	17	1.7

AEI: adverse event of interest; AR: adverse reaction; ESS: enhanced safety surveillance; PRAC: Pharmacovigilance Risk Assessment Committee.

a Intanza 15 µg only given to those >60 years of age.

Twenty-one Intanza 15 µg vaccinees (VRR = 2.1%) reported 103 suspected ARs following vaccination (ARR = 10.3%), with 101 suspected ARs occurring within 7 days of vaccination. Reporting rates observed in the 2016/17 ESS (2.9%) were similar to 2015/16 ESS reporting rates (2.1%) (Table 1).

The most frequently reported preferred terms (PTs) for suspected ARs (n≥3) are provided in Table 2.

**Table 2.** Summary of most frequently (n≥3) reported preferred terms for suspected adverse reactions for Vaxigrip and Intanza 15 µg during 2016/17 enhanced safety surveillance.

Suspected AR Preferred term^a^	n	Percentage	95% CI
Vaxigrip (N = 962)^a^			
Pyrexia	5	0.5	0.1, 1.0
Headache	4	0.4	0.1, 1.1
Malaise	4	0.4	0.1, 1.1
Exposure during pregnancy	3	0.3	0.1, 0.9
No adverse event	3	0.3	0.1, 0.9
Vaccination site erythema	3	0.3	0.1, 0.9
Intanza 15 µg (N = 1000)			
Oropharyngeal pain	8	0.8	0.2, 1.4
Headache	6	0.6	0.1, 1.1
Malaise	6	0.6	0.1, 1.1
Vaccination site erythema	6	0.6	0.1, 1.1
Cough	4	0.4	0.1, 1.0
Rhinorrhoea	4	0.4	0.1, 1.0
Vaccination site swelling	4	0.4	0.1, 1.0
Feeling hot	3	0.3	0.1, 0.9
Influenza like illness	3	0.3	0.1, 0.9
Pain in extremity	3	0.3	0.1, 0.9
Pruritus	3	0.3	0.1, 0.9
Vaccination site inflammation	3	0.3	0.1, 0.9
Vaccination site pain	3	0.3	0.1, 0.9

AR: adverse reaction; CI: confidence interval.

a All ARs with Vaxigrip or Intanza 15 µg were reported within ≤7 days and were non-serious.

In the Vaxigrip group, pyrexia was the most frequently reported PT followed by headache and malaise. Per age group, “No Adverse Event (AE)” and “Exposure during Pregnancy” were the most frequently reported PTs and was expected each with a maximum occurrence of 3 (ARR = 2.1%).

In the Intanza 15 µg group, oropharyngeal pain was the most frequently reported PT, followed by headache, malaise, and vaccination site erythema (Table 1). No suspected ARs occurred at a frequency >1%.

Abasia (inability to walk) and hyperpyrexia (high fever corresponding to 40°C and more) were reported as serious suspected ARs (serious ARR = 0.2%) in addition to decreased appetite, chills, headache and arthralgia in a 4-year-old child 3 days after Vaxigrip vaccination. All events were unresolved at the time of reporting and no medically confirmed diagnosis was provided. The fact that the patient could not walk may have been due to the condition of the patient with high fever and arthralgia. A causative role for Vaxigrip was not excluded.

Three serious suspected ARs (serious ARR = 0.3%) were reported within 7 days after Intanza 15 µg vaccination. An over 60 year-old vaccinee and a 70 year-old vaccinee reported lower respiratory tract infections (resolved and unresolved, respectively). A 68 year-old vaccinee reported a urinary tract infection (outcome not available) in which a causative role for Intanza 15 µg was considered not likely based on the nature of the reported events.

When considering only those adverse events of interest (AEIs) as defined by the PRAC,^2^ 12 Vaxigrip vaccinees (VRR = 1.2%) reported 28 suspected PRAC-defined AEIs (ARR = 2.9%; Table 3). In the Intanza 15 µg group, 17 vaccinees (VRR = 1.7%) reported a total of 44 suspected PRAC-defined AEIs (ARR = 4.4%). Reporting rates for PRAC AEIs for Vaxigrip and Intanza 15 µg from the current ESS are similar to those observed during the 2015/16 ESS (Table 1). All suspected PRAC-defined AEIs occurred within 7 days of vaccination for Intanza 15 µg and Vaxigrip across all age groups (Table 3). No obvious distribution pattern in the type of suspected ARs or in their frequency was observed across the age groups.

**Table 3.** Summary of PRAC adverse reactions of interest by age group, reported in ≥2 vaccinees in the 2016/17 influenza season and comparison with the 2015/16 influenza season.

	2015/16 ESS						2016/17 ESS		
	≤7 days			Total^a^			Total (≤7 days)^a^		
Preferred term	n	%	95% CI	n	%	95% CI	n	%	95% CI
Vaxigrip									
Total number of PRAC AEIs	42	4.1	-	46	4.5	-	28	2.9	-
Total number of vaccinees with PRAC AEIs	22	2.2	1.3, 3.1	25	2.5	1.5, 3.4	12	1.2	0.5, 1.9
6 months to <6 years									
Number of PRAC AEIs	20	4.0	-	21	4.2	-	11	1.1	-
Number of vaccinees with PRAC AEIs	11	2.2	0.9, 3.5	12	2.4	1.1, 3.8	4	3.1	0.8, 7.6
Pyrexia	7	1.4	0.4, 2.4	8	1.6	0.5, 2.7	2	1.5	0.2, 5.4
Decreased appetite	0	0	-	0	0	-	2	1.5	0.2, 5.4
Vaccination site erythema	3	0.6	0.1, 1.8	3	0.6	0.1, 1.8	2	1.5	0.2, 5.4
≥6 years to <13 years									
Number of PRAC AEIs	7	6.3	-	7	6.3	-	8	0.8	-
Number of vaccinees with PRAC AEIs	2	1.8	0.2, 6.4	2	1.8	0.2, 6.4	3	1.6	0.3, 4.5
Pyrexia	1	0.9	0.0, 4.9	1	0.9	0.0, 4.9	2	1.0	0.1, 3.7
Headache	0	0	-	0	0	-	2	1.0	0.1, 3.7
≥13 to <18 years									
Number of PRAC AEIs	No data reported for this age group in the 2015/16 influenza season						No data reported for this age group in the 2016/17 influenza season		
Number of vaccinees with PRAC AEIs									
≥18 to 65 years									
Number of PRAC AEIs	4	2.7	-	4	2.7	-	6	0.6	-
Number of vaccinees with PRAC AEIs	2	1.3	0.2, 4.8	2	1.3	0.2, 4.8	2	1.4	0.2, 4.9
	No AEIs reported for this age group in the 2016/17 influenza season by >1 vaccinee								
>65 years									
Number of PRAC AEIs	11	4.6	-	14	5.9	-	3	0.3	-
Number of vaccinees with PRAC AEIs	7	3.0	0.8, 5.1	9	3.8	1.4, 6.2	3	0.7	0.2, 2.1
	No AEIs reported for this age group in the 2016/17 influenza season by >1 vaccinee								
Intanza 15 µg^b^									
Total number of PRAC AEIs	53	5.2	-	56	5.5	-	44	4.4	-
Total number of vaccinees with PRAC AEIs	26	2.6	1.6, 3.5	28	2.8	1.7, 3.8	17	1.7	0.9, 2.5
Vaccination site erythema	9	0.9	0.3, 1.5	9	0.9	0.3, 1.5	6	0.6	0.1, 1.1
Headache	2	0.2	0.0, 0.7	3	0.3	0.1, 0.9	6	0.6	0.1, 1.1
Malaise	6	0.6	0.1, 1.1	7	0.7	0.2, 1.2	6	0.6	0.1, 1.1
Vaccination site swelling	5	0.5	0.1, 0.9	5	0.5	0.1, 0.9	4	0.4	0.1, 1.0
Vaccination site inflammation	0	0	-	0	0	-	3	0.3	0.1, 0.9
Vaccination site pain	10	1.0	0.4, 1.6	10	1.0	0.4, 1.6	3	0.3	0.1, 0.9
Localised oedema	0	0	-	0	0	-	2	0.2	0.0, 0.7
Vaccination site pruritus	5	0.5	0.1, 0.9	5	0.5	0.1, 0.9	2	0.2	0.0, 0.7
Vaccination site vesicles	0	0	-	0	0	-	2	0.2	0.0, 0.7
Pyrexia	2	0.2	0.0, 0.7	2	0.2	0.0, 0.7	2	0.2	0.0, 0.7

AEI: adverse event of interest; CI: confidence interval; PRAC: pharmacovigilance risk assessment committee

Note: PRAC AEIs as listed in the guidance were specifically described and are as follows: injection-site reactions (pain, erythema, pruritus, swelling, induration, and ecchymosis) and systemic reactions (fever [>38°C], headache, malaise, myalgia, shivering, rash, vomiting, nausea, arthralgia, decreased appetite, irritability [for vaccinees <5 years old], crying [for vaccinees <5 years old], and events indicative of allergic and hypersensitivity reactions, including ocular symptoms) [2].

a Total refers to the PRAC AEIs by age group reported in ≥2 vaccinees in the 2015/16 influenza season within 7 days and >7 days. In 2016/17 influenza season, all PRAC AEIs occurred within 7 days of vaccination for Intanza 15 µg and Vaxigrip across all age groups.

b Intanza 15 µg only given to those >60 years of age.

Following Vaxigrip vaccination, vaccinees reported 3 mild, 1 moderate, and 5 severe suspected PRAC-defined AEIs. Severity was unknown for the remaining 19 suspected PRAC-defined AEIs. Following Intanza 15 µg vaccination, vaccinees reported 29 mild, 3 moderate, none severe, and 12 suspected PRAC-defined AEIs of unknown severity.

## Comparison of the reported frequencies of the 2016/2017 ESS with the 2015/16 ESS reference data and summary of product characteristics

### Vaxigrip

None of the observed frequencies of suspected ARs or PRAC-defined suspected AEIs during the 2016/17 ESS were above those of 2015/16 ESS. Additionally, there were no ‘Other suspected ARs’ that could be compared with the Summary of Product Characteristics (SmPC). Abasia is listed as one of these ‘other’ ARs, however, abasia is not expected with Vaxigrip and therefore cannot be compared with the SmPC.

### Intanza 15 µg

The AR “oropharyngeal pain”, reported within 7 days (n = 8, VRR = 0.8%, 95% CI: 0.2%-1.4%) was the only individual PT that crossed the upper limit of the historical threshold based on the results of the 2015/16 ESS (VRR = 0.2%, 95% CI: 0.0%-0.7%). All 8 vaccinees who reported “oropharyngeal pain” also reported concomitant AEs compatible with cold symptoms (for instance, chest discomfort in 2 cases or cough in 3 cases). Therefore, a role of the inactivated influenza vaccine is unlikely.

None of the observed frequencies for PRAC-defined suspected AEIs during the 2016/17 ESS were above those of the 2015/16 ESS. Two ‘other ARs’ (fatigue and paraesthesia) were listed in the SmPC and could be compared by frequency: The reported frequency of fatigue (0.2%) is consistent with the frequency reported in the SmPC whereas paraesthesia (0.2%) was higher than that reported in the SmPC based on the 2 cases that occurred within 7 days of Intanza 15 µg vaccination. Paraesthesia resolved in one vaccinee (68 year-old) but remained unresolved in the other vaccinee (>60 year-old).

## Discussion

The current passive ESS was implemented with some changes with reference to the 2015/16 ESS. These changes were made to improve operational efficiency. Several considerations were taken into account to identify the best countries to conduct the current ESS. The countries chosen were those with higher influenza vaccination coverage and the potential to capture paediatric vaccination in the absence of universal recommendation for influenza vaccination in Europe. Countries with a process of annual tender, which determines yearly the influenza vaccines available on the market for the next influenza season, and with late tender results for awarding a specific influenza vaccine were excluded. Knowing late the availability of Vaxigrip or Intanza 15 µg in a country for the upcoming season makes it difficult to initiate the ESS in this country notably in terms of Ethics Committee approvals and sites selection/ initiation. In addition, countries in which the ESS was successfully conducted during the previous year were also considered. Therefore, the selected countries were not necessarily the first to use Vaxigrip or Intanza 15 µg vaccine doses in Europe. The UK was selected for this ESS because the UK possessed pre-identified sites that would use either Intanza 15 µg or Vaxigrip during 2016/17 season. Nevertheless, since there is a preferential recommendation in the UK to vaccinate all children from 2 to 8 years of age and to children from 2 to 18 years of age in clinical risks groups with the live attenuated influenza vaccine (unless contraindicated), paediatric age group was under-represented. Thus, additional sites in the ROI were selected to distribute SRCs only to paediatric vaccinees in view of national recommendations for childhood influenza vaccination to increase the representation of this population in the ESS. In 2015/16 ESS, Finland also distributed only paediatric SRCs, however, most of the SRCs (n = 496) were distributed to vaccinees between 6 months and ≤6 years of age and were distributed later in the season since the influenza vaccination season started at the beginning of November in Finland.

The strengths of the 2016/2017 ESS were that in the UK and ROI, HCPs were pre-selected based on their influenza vaccination capacities, ensuring sufficient age group representation in the total vaccinated population. Lessons learned from 2015/16 ESS were leveraged in the UK site, including consideration of the site's vaccination clinic start dates, enrolment rates from the 2015/16 ESS, and willingness to participate. Fewer sites were used during the 2016/17 ESS (4 sites) than for the 2015/16 ESS (14 sites). Reduction in the number of sites was conducted in an effort to accelerate vaccinee recruitment. As a result, the 2016/20178 ESS was executed in a more timely manner, starting as early as possible in the season, from 20 September in 2016/17 ESS versus mid-October in 2015/16 ESS. Distribution of the targeted number of SRCs was also more rapid in the 2016/2017 ESS, Twelve days and 39 days in 2016/17 ESS versus 53 and 51 days in the 2015/16 ESS for Intanza 15 µg and Vaxigrip, respectively.

A limitation of the study was that the target to distribute 1000 Vaxigrip SRCs was not reached due to difficulty in recruiting paediatric vaccinees in the ROI, which might have been due to local customs regarding the influenza vaccination process. In the UK, large vaccination clinics are generally advertised in advance to vaccinees registered in practices and are held specifically for influenza vaccination. In contrast, in the ROI, there are no influenza-specific vaccination clinics for at risk children and vaccine is often delivered through opportunity during routinely scheduled appointments in the hospital and GP setting. Hence, the overall observed recruitment number in the ROI (131 vaccinees in age group ≥6 months to <6 years) were lower compared to the 2015/2016 ESS conducted in Finland (488 vaccinees in age group ≥6 months to <6 years). In addition, some Irish sites experienced cancellation of appointments and/or vaccinations for seasonal influenza vaccination due to high levels of paediatric respiratory infections possibly due to the beginning of school term during a warm autumn, and the negative impact of adverse publicity surrounding vaccination in general. Some efficiency may have been lost due to the selection of the ROI over Finland since the 2015/16 ESS experience could not be leveraged. However, selection of the ROI allowed for SRC distribution to begin earlier in the influenza season. Furthermore, in Finland, influenza vaccination of the pediatric population is managed at well-baby clinics. Centralization of influenza vaccination is helpful for this type of ESS. When an ESS is conducted in several countries to ensure a well-balanced representation of age groups for a specific brand, flexibility in SRC distribution between countries is key to ensure the distribution target is reached in a timely manner.

Vaccinees were encouraged to report suspected ARs with an emphasis on those occurring within 7 days following vaccination using contact information found on the SRC. Although HCPs encouraged vaccinees to report any suspected AR, reporting remained vaccinee-driven and spontaneous in nature. Both the 2016/17 and 2015/16 ESS showed higher AR reporting rates than previous spontaneous reporting.^4^ Historically, spontaneous reporting rates after seasonal influenza vaccination have been generally low and ranged from 20 to 90 reports per 1,000,000 people vaccinated.^5–9^ Passive ESS was shown to increase reporting rates from 2- to 5-fold in an Australian^10^ and an Italian study.^11^

In the 2016/17 ESS, we observed an overall VRR of 1.8% and an ARR of 6.1% for Vaxigrip. These reporting rates are slightly lower than those observed in 2015/16 ESS (VRR = 3.2% and ARR = 12.1%) which could be due to the fact that the percentage of Vaxigrip SRCs distributed across age groups differed between the two years. This may have affected number and types of ARs received, particularly in the 6 mo. to <6 yr and >65 years age groups. Within age groups, the largest ARR decrease was observed in those >65 years of age. The 2015/16 ESS had an ARR of 21.1% due, in part, to a single vaccinee who reported 13 suspected ARs within 7 days, whereas the 2016/17 ESS had an ARR of 4.1%. In 2016/17 ESS, the highest ARR (13.0%) was reported by 4 vaccinees within 7 days in children 6 months to <6 years (expected in this age group), although it was slightly higher than the 2015/16 ESS (8.1%). For Intanza 15 µg, an overall VRR of 2.1% and an ARR of 10.1% were observed, which were similar to 2015/16 ESS (VRR = 3.1% and ARR = 11.2%).

During the 2016/17 ESS, all 5 age groups were represented for Vaxigrip. Similar to the 2015/16 ESS, the lowest representation was in the 13 to 18 yr age group. While during the 2016/17 ESS more SRCs were distributed to this age group (8.8% of the population), the results indicate that this specific age group is difficult to capture. Consequently, in the ROI only 397 of the 500 intended SRCs for paediatrics were distributed. For Intanza 15 µg, adult age group was split as per the guidance for the 2015/16 ESS (adults from 18 to 65 years and adults ≥65 years). However, since Intanza 15 µg is indicated in individuals ≥60 years of age, this split in age groups was not applied in this year's ESS as the overall reporting rate would be more meaningful in this age group. No clinically significant changes compared to what is known or expected with Vaxigrip or Intanza 15 µg (product information of the vaccines and previous ESS results) was observed during the 2016/17 ESS.

The EMA recommends that the analysis of more than one batch be conducted to better evaluate a safety signal.^2^ For the 2016/17 ESS, although more than one batch of Intanza 15 µg and Vaxigrip was used by the selected sites, nearly all Intanza 15 µg patients received the same batch in routine vaccination. The 2016/2017 ESS provided additional but limited safety data on the seasonal influenza vaccines. Similar methodology with efforts to improve age group representation, an early start and more rapid data collection was employed in the 2016/2017 ESS. Reporting rates were similar to those observed during the 2015/2016 season. With the intent to build a sustainable ESS system in Europe, discussions should take place at the European level involving all stakeholders to share experiences and expectations, to analyze the value of current ESS output for the regulators, and to develop a more integrated system that could allow ESS expansion to several countries during a specific season. Proposals such as improved integration of the ESS PV reporting into the existing routine PV system, or improved documentation of specific brand use in routine practice and increased awareness on the need for more systematic reporting of any suspected ARs should be discussed. A close follow-up of other European initiatives will be important for future improvements of the ESS, such as the ADVANCE IMI (Accelerated Development of Vaccine Benefit-Risk Collaboration in Europe -Innovative Medicines Initiative) project^12^ outputs that would provide a framework for future public-private partnership for vaccine benefit/risk monitoring; or DRIVE (Development of Robust and Innovative Vaccine Effectiveness) IMI project that aims to create a platform under a public-private partnership with the capacity to perform influenza vaccine effectiveness assessments.

Enhanced safety monitoring may contribute to an increased awareness and confidence in vaccine safety. Based on the experience gained from two seasons of passive ESS, the methodology proposed may represent a suitable step for enhancing the surveillance of seasonal influenza vaccines early in the influenza season.

## Materials and methods

### Study design

This was a multicenter, non-interventional, observational, passive ESS conducted in two European countries, the UK and the ROI. Near real-time data were collected and analyzed as described previously.^3^

### Setting

The start of the current ESS coincided with the start of routine influenza vaccination for the 2016/17 influenza season by the selected HCPs, i.e., 20 September 2016 in the UK and 21 September 2016 in the ROI [Vaxigrip only]). The study ended when 1000 SRCs per influenza vaccine brand were distributed + 2 weeks to allow vaccinee reporting or 2 months (6 weeks for SRC distribution + 2 weeks for vaccinee reporting) following the distribution of the first SRC, whichever came first.

### Participants

Vaccinees who received either Vaxigrip (indicated for persons 6 months of age and older) or Intanza 15 µg (indicated for persons 60 years of age and older (UK only) in routine practice and according to the national recommendation for influenza vaccination, who also received an SRC were eligible for participation in this study (ESS population). In the ROI, inclusion of vaccinees who received Vaxigrip was limited to those aged ≥6 months to <18 years to ensure adequate paediatric representation.

## Procedures and data collection method

Paper SRCs specific to Vaxigrip or Intanza 15 µg were distributed to vaccinees and provided instructions on how to report suspected ARs. The detailed procedures and data collection methods were similar to those previously published.^3^

All events were reported spontaneously by vaccinees or HCPs and thus considered suspected ARs unless the reporters specifically stated that they believed the events to be unrelated or that a causal relationship could be excluded. No causality assessment was requested from the vaccinee or HCPs or performed by the Marketing Authorization Holder for these cases. All suspected ARs were categorized and summarized by brand and age group as follows: PRAC-defined adverse events of interest (AEIs) as per guidance,^2^ suspected ARs classified as “identified or potential risks” based on individual risk management plans, and other suspected ARs, i.e., those that were not classified under above categories. Safety signals were defined per Good Pharmacovigilance Practice Annex I revision 3.^13^

### Population size

A total of 1000 SRCs per vaccine brand were targeted for distribution to enable the detection of ARs expected to be common.^2^ The number of vaccinees who would potentially report suspected ARs could be stimulated but not controlled.

### Bias

No potential sources of bias were identified for this safety surveillance. However, some degree of selection bias might have occurred in the ESS. For example, vaccinees who accepted the SRC might have reported more or fewer ARs than those who refused the SRC, or the HCP may have proposed the SRC to select vaccinees, which could have influenced the reporting rate.

### Statistical analysis

All analyses were descriptive and produced using SAS version 9.2 (SAS Institute, Cary, North Carolina). Verbatim ARs were coded with Medical Dictionary for Regulatory Activities terminology (version 19.0). No confirmatory hypothesis testing was conducted for the analyses.

The frequency and percentage of VRR (primary endpoint) and ARR were presented according to their PTs and system organ class (SOC).

$$\text{Vaccinees' reporting rate}\left(\text{VRR}\right)=\frac{N\text{umber}\;\text{of}\;\text{vaccinees}\;\text{who}\;\text{reported}\;\text{atleast}\;\text{one}\;\text{AR}}{Total\;number\;of\;SRCs\;\;distributed}\times \;\text{100}$$

$$\text{AR reporting rate}\left(\text{ARR}\right)=\frac{N\text{umber}\;\text{of}\;\text{suspected}\;\text{ARs}}{Total\;number\;of\;SRCs\;\;distributed}\times \;\text{100}$$

For VRR, two-sided 95% confidence intervals (CIs) were calculated using the Wald method if the number of vaccinees who reported ≥1 AR was ≥5 otherwise the exact method was used (number of vaccinees who reported ≥1 AR being <5).

All VRRs and ARRs were estimated by age groups (for Vaxigrip: ≥6 months to <6 years, ≥6 to <13 years, ≥13 to <18 years, ≥18 to ≤65 years, and >65 years; for Intanza 15 µg: ≥60 years), seriousness (yes/no), severity (mild, moderate, severe, or unknown as per protocol), by day of onset since vaccination (≤7, >7 days), and per brand in total. The suspected ARRs and VRRs from the 2016/17 ESS were compared with the reporting rates from the 2015/16 ESS.

### Ethics

The ESS was conducted in accordance with Good Epidemiological Practice and the European Network of Centres for Pharmacoepidemiology and Pharmacovigilance.^14-16^ The ESS was submitted to country authorities as defined by local regulations, and local ethics committee approvals were obtained. The ESS relies on routine pharmacovigilance and enhanced voluntary spontaneous reporting. From this point of view, no informed consent was required. The vaccinee or parent/legal guardian (it was not expected that children or adolescents would call the Contact Support Centre) provided “implied consent” on two occasions: first, when he/she accepted the SRC; second, when he/she decided to call the Contact Support Centre. In addition, the vaccinee or parent/legal guardian provided consent to record the call. All procedures were performed according to the protocol and documented appropriately.

## Conclusions

The reported suspected ARs and the frequency observed are consistent with the safety profiles of Vaxigrip and Intanza 15 µg. The data did not show any change in reactogenicity of Vaxigrip or Intanza 15 µg or any other safety concern as compared to what is known or expected (product information and previous season ESS results). ESS allows generation of early safety data that could reduce the safety concerns of the general population with regard to vaccines in general and influenza vaccines in particular. The experiences gained from 2015/16 and 2016/17 ESS will help in improving the execution of passive 2017/18 ESS early in the season.

## Trademark statement

Vaxigrip and Intanza 15 µg are registered trademarks of Sanofi Pasteur.
